# Knockdown of *NtCPS2* promotes plant growth and reduces drought tolerance in *Nicotiana tabacum*

**DOI:** 10.3389/fpls.2022.968738

**Published:** 2022-11-08

**Authors:** Shixiao Xu, Wenlong Han, Kexin Cao, Bo Li, Cong Zheng, Ke Xie, Wei Li, Lingxiao He

**Affiliations:** ^1^ Henan Agricultural University, College Tobacco Science, National Tobacco Cultivation & Physiology & Biochemistry Research Center, Scientific Observation and Experiment Station of Henan, Ministry of Agriculture, Zhengzhou, Henan, China; ^2^ China Tobacco Zhejiang Industry Co, Ltd., Hangzhou, China; ^3^ Fujian Tobacco Corporation Nanping Company, Nanping, Fujian, China; ^4^ College of Agronomy, Sichuan Agricultural University & Sichuan Engineering Research Center for Crop Strip Intercropping System & Key Laboratory of Crop Ecophysiology and Farming System in Southwest, Ministry of Agriculture, Chengdu, Sichuan, China

**Keywords:** drought stress, *NtCPS2*-knockdown, gibberellin, abscisic acid, genome editing

## Abstract

Drought stress is one of the primary environmental stress factors that gravely threaten crop growth, development, and yields. After drought stress, plants can regulate the content and proportion of various hormones to adjust their growth and development, and in some cases to minimize the adverse effects of drought stress. In our previous study, the tobacco *cis*-abienol synthesis gene (*NtCPS2*) was found to affect hormone synthesis in tobacco plants. Unfortunately, the role of *NtCPS2* genes in the response to abiotic stress has not yet been investigated. Here, we present data supporting the role of *NtCPS2* genes in drought stress and the possible underlying molecular mechanisms. *NtCPS2* gene expression was induced by polyethylene glycol, high-temperature, and virus treatments. The results of subcellular localization showed that *NtCPS2* was localized in the cell membrane. The *NtCPS2*-knockdown plants exhibited higher levels of gibberellin (GA) content and synthesis pathway genes expression but lower abscisic acid (ABA) content and synthesis pathway genes expression in response to drought stress. In addition, the transgenic tobacco lines showed higher leaf water loss and electrolyte loss, lower soluble protein and reactive oxygen species content (ROS), and lower antioxidant enzyme activity after drought treatment compared to wild type plants (WT). In summary, *NtCPS2* positively regulates drought stress tolerance possibly by modulating the ratio of GA to ABA, which was confirmed by evidence of related phenotypic and physiological indicators. This study may provide evidence for the feedback regulation of hormone to abiotic and biotic stresses.

## Introduction

*cis*-Abienol belongs to labdane terpenoids, which occur widely in Abies oleoresins and in particular in the oleoresin of *Abies balsamea* (L.) Mill. (Canada balsam) (Carman and Dennis, 1968). At present, all the abienol reported are *cis*-abienol, but the existence of *trans*-abienol has not been reported in nature (Jassbi et al., 2017). Labdane terpenoids were first isolated from aged Turkish tobacco by Giles and Schumacher (1961), and *cis*-Abienol was the main labdane terpenoid in tobacco. *cis*-Abienol is one of the terpenoid aroma precursors peculiar to the trichome exudate of most aromatic tobacco and some cigars (Wang, 2016), which is synthesized by the drive of 8-hydroxy-Copalyl diphosphate synthase (CPS2) by trichome specific promoter (Philipp et al., 2012). Sallaud et al. (2012) proved through *in vitro* experiments that the *NtCPS2* gene encodes cobacyl pyrophase synthase and participates in the synthesis of 8-hydroxy-cobacyl pyrophophate (8-OH-CPP), a precursor of labdane compounds. Zhang et al. (2020) used CRISPR/Cas9 technology to knock out the *CPS2* gene in tobacco, and the results showed that the expression level of *CPS2* and the content of abienol were significantly reduced, so the synthesis of *cis*-abienol compound could be inhibited after knocking out *CPS2*.

Isoprenoids are essential for normal growth and development processes in all living organisms (Pulido et al., 2012). Isopentenyl diphosphate (IPP; C5) is a common metabolic precursor of all isoprenoids (Engprasert et al., 2004). It has been reported that there are two distinct pathways of IPP synthesis in plants, one being the mevalonate (MVA) pathway in the cytoplasm and the other being the alternative mevalonate-independent (2C-methyl-D-erythritol 4-phosphate; MEP) in the plastids (Rohmer et al., 1993; Rodríguez-Concepción and Boronat, 2015; Barja and Rodríguez-Concepción, 2021). Geranylgeranyl diphosphate (GGPP) synthase catalyses the consecutive condensation of an allylic diphosphate with three molecules of IPP to produce GGPP, an essential linear precursor for the biosynthesis of terpenoids (Wang and Ohnuma, 1999). *cis*-Abienol, and gibberellin (GA) belong to diterpenoids and carotenoids belong to tetraterpenoids, which were synthesized *via* a non-mevalonate pathway (Rohmer et al., 1993). GGPP forms the precursor substance 8-OH-CPP of *cis*-abienol, the precursor substance copalyl-pyrophophate (CPP) of GAs and the precursor substance carotenoid of abscisic acid(ABA)under the catalytic action of CPS2, *ent*-copalyl diphosphate synthase (CPS) and phytoene synthase(PSY)respectively (Rohmer et al., 1993; Wang et al., 2001; Cheng et al., 2002; González-guzmá et al., 2002; Sallaud et al., 2012; Finkelstein, 2013).

Recently, we performed transcriptome analyses of tobacco plants with *cis*-abienol synthesis gene *NtCPS2* knockdown and studied their physiology and biochemistry characteristics (He et al., 2021a; He et al., 2021b). We found that knocking down the *cis*-abienol synthesis gene *NtCPS2* not only reduced the relative expression of *NtCPS2* gene and the content of *cis*-abienol, but also affected related genes in the GA synthesis pathway, thus increasing the GA content. In addition, it was reported that ABA and GA interact with each other and regulate plant growth, such as seed germination and dormancy (Tang et al., 2021), and plant shoot growth (Qin et al., 2013; Shu et al., 2018). Yang et al., 2015 found the activities of MDA, Vit C, Proline and CAT in *Ziziphus jujuba* Mill. treated with exogenous ABA and GA3 of different concentrations were characterized by mutual restriction or antagonism. Song et al. (1998) showed that GA3 and ABA had antagonistic effects, and the intensity of antagonistic effects varied with different semi-dwarf varieties of indica rice. Moreover, under the condition of gibberellin treatment, the inhibition of ABA on shoot growth of rice was relieved (Shu et al., 2018).

ABA regulates stomatal movement (Cutler et al., 2010; Hubbard et al., 2010; Popko et al., 2010; McAdam and Brodribb, 2015; Wilkinson and Davies, 2010), while stomata regulation is one of the most important mechanisms that enable plants to regulate and optimize water loss through evaporation (Chaves et al., 2016). In angiosperms, ABA binds to receptors (plasma membrane receptors and intracellular receptors) to activate G proteins, which in turn activate phospholipase, resulting in abi1^-1^, NAD (P) H-dependent ROS and ABI2^-1^ involved in Ca^2+^ signal transduction (Murata et al., 2001). Through Ca^2+^ dependent signal transduction pathways, Ca^2+^ inhibits K^+^ internal circulation channels, activates K^+^ outflow channels and anionic channels, and promotes K^+^ outflow (Grabov and Blatt, 1998a; Grabov and Blatt, 1998b; Macrobbie, 2000), ultimately leading to stomatal closure or inhibiting stomatal opening (Lemichez et al., 2001). In addition, ABA induces stomatal closure through Ca^2+^ independent signal transduction pathways, i.e. by raising cytoplasmic pH and activating K^+^ outflow channels and anionic channels (Hartung et al., 1998; Netting, 2000). The stomata of leaves are the main channels for plant water loss (Lin et al., 2020). Under conditions where soil water is limited and/or there is a high atmospheric evaporative demand, the stomata on the leaf surface close partially or completely depending on the turgidity of the surrounding guard cells to maintain a favourable water balance while limiting carbon gain (Boyer, 1982; Ciais et al., 2005; Franks, 2013; Vinya et al., 2013). Tombesi et al. (2015) have shown that in *V. vinifera* passive hydraulic control of stomatal closure appears to be dominant over any chemical signals during the early stages of drought stress. (Dow et al. 2014a; Dow et al., 2014b) showed that the number, size, and distribution of stomata (stomatal trait) influence stomatal conductance. The stomatal pores on the leaf surface open or close depending on the severity of the surrounding guard cells to protect against desiccation. (Gupta et al., 2020).

At present, the effects of *NtCPS2* on the diterpenoid metabolic synthesis pathway have been reported (Philipp et al., 2012; Sallaud et al., 2012), but the relationship between the *NtCPS2* gene and abiotic - biotic stress is rarely reported. The flue-cured tobacco strain ‘8306’ was used in this study. We compared the physiological and biochemical differences of wild-type (WT) and transformed *NtCPS2*-knock down plants (T_3_-26, T_3_-45, T_3_-48) under abiotic or biotic stress, especially drought stress, to clarify the regulation mode of *NtCPS2* gene on abiotic and biotic stresses and provide evidence for the feedback regulation of terpenoids to abiotic and biotic stresses.

## Materials and methods

### Plant materials, growth conditions, and experimental treatments

Seeds from the wild-type (WT) and the homozygous transgenic plants T2 generation (T_3_-26, T_3_-45 and T_3_-48) were collected and grown hydroponically in a growth room with 65-70% relative humidity, 28 ± 2°C daytime and 18 ± 2°C nighttime temperatures, a photoperiod of 14 h light/10 h darkness at a light intensity of approximately 4000 Lx. Seeds were first germinated in an I-shaped square seedling sponge with Hoagland’s solution. The germinated seeds were then transferred to vermiculite for growth. Tobacco plant seedlings with three true leaves were transplanted into hydroponic plastic pots for the experiments.

For poly-ethylene glycol (PEG-6000) treatments, 10-week-old seedlings were transferred to Hoagland’s nutrient solution supplemented with 0% PEG-6000 (Control) and 20% PEG-6000 (Drought) for cultivation. And seedlings were sampled separately after PEG-6000 treatment for 6h, and plant phenotypes were recorded. These experiments were performed in three biological replicates. Harvested samples were immediately frozen in liquid nitrogen and then stored at −80°C for subsequent experiments.

For heat treatments, 10-week-old seedlings were maintained at 45°C for heat stress (65% relative humidity) in growth chambers, and seedlings were sampled separately after heat treatment (0, 48h), and plant phenotypes were recorded. And fresh PVY-infected or TMV-infected tobacco leaves were frozen in liquid nitrogen and thoroughly crushed, then diluted 1:100 with water (Ren et al., 2021). The transgenic and WT plants were inoculated with PVY or TMV by rubbing, and susceptibility symptoms were assessed after 5 or 7 days. These experiments were performed in ten biological replicates. Harvested samples were immediately frozen in liquid nitrogen and then stored at −80°C for RNA isolation.

### CRISPR/Cas9 construction strategy and vector information

The gene knockdown model of *NtCPS2* was constructed using CRISPR/Cas9 gene editing technology in tobacco (*Nicotiana tabacum* cv. 8306) to inhibit the function of *NtCPS2 in vitro* (Zhang et al., 2020). Based on the provided mRNA sequences and corresponding genomic sequence information, 2 CRISPR target sites (**Table S1**) were designed to improve the gene targeting efficiency. The target loci PCR amplification primers were designed (**Table S2**), and the primers were sent to Tianyi Huiyuan Biotechnology Co. for synthesis. After primer synthesis, the fragment containing the target site was amplified by overlap extension PCR. PCR fragment was cloned into the final CRISPR expression vector using recombinase from Nanjing Novozymes Biotechnology Co. The constructed CRISPR vector was electrotransferred into E. coli and positive clones were screened based on colony PCR for transformation of Agrobacterium and genetic transformation of tobacco strains.

Mutation detection of target gene target site sequences in edited plants

Tobacco genomic DNA was extracted by a modified CTAB method. samples T1-26, T1-45, and T1-48 were ground using a grinder, and then genomic DNA was extracted using a CTAB extraction buffer. DNA samples were air-dried and added to ddH2O for 100 μL.

The primers 17 KN48-df (5’→3’): ATCATAGCGGAATTGTTTGTCTC and 17 KN48-dr (5’→3’): TCCGTATAGATACCTAAGCGATCTG were designed to amplify the target bands and purified. The sequencing results were compared with the template sequences using DANMAN 6.0 software. Amplification reaction system: 2×PCR Mix10μL each primer 0.3μL, ddH_2_O 8. 9μl, tobacco genome dna0.5μl. reaction procedure: 94°C pre-denaturation 5 minutes, 32 cycles (94°C30s, 56°C30s, 72°C40s), 72°C5 minutes, 25°C1 minute. The above PCR amplification products were detected by electrophoresis using 1% lipose gel.

The relative expression of Nicotiana tabacum ribosomal protein L25 (L25, L18908) was stable across treatments, tissues, and periods and was suitable for use as an internal reference. Schmidt et al. evaluated the expression stability of eight commonly used tobacco internal reference genes based on 22 samples of K326, which were in descending order of L25 > EF-Ia > Ntubc2 > PP2A > 18SrRNA > Actin > B-Tubulin > a-Tubulin (Schmidt and Delaney, 2010).

Four promoter sequences (pGhU6.1, pGhU6.4, pGhU6.7, and pGhU6.9) were amplified from the tobacco genome and verified with Sanger sequencing. Promoter pGhU6.9 was finally chosen for vector modification because of its high identity with that of AtU6-26. Its Bsa I restriction site was mutated to avoid multiple Bsa I sites in the final vector. The pRGEB32 plasmid, a gift from Xie et al. (2015), was linearized with Hind III and Sbf I double digestion, resulting in deletion of the gRNA terminator fragment. The promoter pGhU6.9 was assembled with the gRNA-terminator segment using an overlapping PCR method. The assembled fragment was inserted to the linearized pRGEB32 using ClonExpressII One Step Cloning Kit (Vazyme, Nanjing, China), thus generating the pRGEB32-GhU6.9 vector. It has a hpt selection marker and was used to target a reporter gene DsRed2, which was firstly transformed into cotton with a vector carrying NPT II (Neomycin phosphotransferase II). For endogenous gene targeting, we changed the selection marker hpt with NPT II between two restriction sites, Pspx I and Xmal I, using the abovementioned one-step cloning strategy. This vector, defined as pRGEB32-GhU6.9-NPT II, was used for stable genetic transformation to knock out NtCPS2 in tobacco (**Figure S1**).

### Subcellular localization analysis

To verify the subcellular localization of the CPS2 protein, the coding sequence of CPS2 was cloned into the GFP vector (pHBT-GFP-NOS) driven by the 35S promoter, to generate the CPS2-GFP fusion protein (**Figure S2**). The resulting vector was then transformed into protoplasts of *Nicotiana benthamiana* following a previously published protocol (Liu et al., 2017; Xu et al., 2020). Afterward, the fluorescence signals of the protoplasts were observed using a confocal laser scanning microscope (Lecia sp8, Germany).

### Seeds germination characteristics and plant biomass

The moistened and sterilized absorbent cotton and filter paper were soaked with deionized water in a clean petri dish, then laid on the petri dish. 100 disinfected seeds were evenly ordered on the surface filter paper and cultured in an artificial climate incubator (MGC-350BP-2L, Yiheng, China). The light intensity was set at 4950 LX, the light duration was 12 h/d, the relative humidity was 60%, and the temperature was 26°C.Timed observations were made every day, and deionized water was added in time. The number of germinated seeds was counted on 7d (germination potential) and 14d (germination rate) after sowing, and the germinating standard was the radicle exceeding 1/2 of the seed length (Ye et al., 2017). Plant biomass measurements included fresh weight. The leaf fresh weight of the plant was measured 70d after seeding.


Germination potential=(number of seeds germinated after 7 days of cultivation/number of seeds tested)×100%

Germination rate=(number of seeds germinated after 14 days of cultivation/number of seeds tested)×100%

### Water loss rate and stomatal apertures analyses

For water loss rate studies, the leaves of 10-week-old transgenic and WT plants were detached, placed on dry philtre paper at room temperature, and weighed at time points (0,0.5, 1, 1.5, 2, 2.5 and 3 h). The water loss rates were determined on the basis of the water content of the leaves. Water loss rates were calculated based on the initial fresh weight of the plants (Zhao et al., 2018).

To understand whether leaf water loss rate mediated by *NtCPS2* is related to stomata movement, leaves were detached from 10-week-old transgenic and WT plants, placed on dry philter paper at room temperature, and stomata were observed with a metalloscope (ML10, Mingmei, China) and stomata rate calculated at time points 0 and 3 h. The stomata size was determined by the stomata movement. The size of the stomata that were smaller than 0.5 µm was considered as closure.

### Measurements of physiological-biochemical parameters

Leaf samples of WT and T_3_-26, T_3_-45, and T_3_-48 were sampled immediately after PEG-6000 treatment for 6h. Leaf samples were measured for relative electrolytic leakage (Zhang et al., 2019), soluble protein (sPRO), hydrogen peroxide (H_2_O_2_) and activities of major antioxidant enzymes, including catalase (CAT), and peroxidase (POD), as described previously. H_2_O_2_ accumulation was detected by 3,3’-diaminobenzidine (DAB) staining as described by Sun et al. (2019). The antioxidant enzymes sPRO, H_2_O_2_, MDA, CAT and POD content or activities were measured using the corresponding detection kits (PC0010, BC3595, BC0025, BC0205, and BC0095, Solarbio, China) following the manufacturer’s protocols. The experiment was repeated at least three times.

### Extraction and assay of phytohormone abscisic acid and gibberellin

ABA and GA in tobacco leaves were determined at different time points (0, 6 h) during PEG-6000 treatment using an ELISA kit provided by China Agricultural University and included three independent biological replicates as described by Chen et al. (2021). The detection limit of quantitative GA in the assay kit is 2–313 ng·mL−1, and that of ABA is 2–311 ng·mL−1. All samples were rapidly frozen in liquid nitrogen and stored at −80 °C prior to analysis.

The extraction, purification and determination of endogenous levels of GA3, ABA and iP + iPA by an indirect ELISA technique were performed as described by He (1993). The samples were homogenised in liquid nitrogen and extracted in cold 80% (v/v) methanol with butylated hydroxytoluene (1 mmol·L−1) overnight at 4°C. The extracts were collected after centrifugation at 10000 × g (4°C) for 20 min, the extracts were passed through a C18 Sep-Pak catridge (Waters, Milford, MA) and dried in N_2_. The residues were dissolved in PBS (0.01 mol·L−1, p H 7.4) in order to determine the levels of GA3, ABA and iP + iPA. Microtitration plates (Nunc) were coated with synthetic GA3, iP + iPA, or ABA-ovalbumin conjugates in NaHCO3 buffer (50 mmol·L−1, pH 9.6) and left overnight at 37°C. Ovalbumin solution (10 mg/ml) was added to each well in order to block nonspecific binding. After incubation for 30 min at 37°C, standard GA3, ABA, iP + iPA, samples and antibodies were added and incubated for a further 45 min at 37°C. The antibodies against GA3, ABA and iP + iPA were obtained as described by Weiler et al. (1981). Then horseradish peroxidase-labelled goat antirabbit immunoglobulin was added to each well and incubated for 1 h a t 37°C. Finally, the buffered enzyme substrate (orthophenylenediamino) was added, and the enzyme reaction was carried out in the dark at 37°C for 15 min, then terminated using 3 mol·L−1 H_2_SO_4_. The absorbance was recorded at 490 nm. Calculations of the enzyme-immunoassay data were performed as described by Weiler et al. (1981). In this study, the percentage recovery of each hormone was calculated by adding known amounts of standard hormone to a split extract. Percentage recoveries were all above 90%, and all sample extract dilution curves paralleled the standard curves, indicating the absence of nonspecific inhibitors in the extracts.

### Determination of photosynthetic parameters

Leaf transpiration rate (E) and stomatal conductance (GS) were measured in the culture chamber using a CIRAS-3 portable photosynthetic system (PP-Systems, UK). Environmental conditions were tightly controlled, with light intensity (PAR) set at 1000μmol/(m^2^·s), molar content of CO_2_ at 390μmol/mol, and temperature at 25°C. The average value of 10 consecutive cycles was taken as the test value. Five representative plants were selected for each variety, and each plant was repeated for three times to get its average value.

### Real-time quantitative PCR

Extraction of total RNAs and synthesis of cDNAs were performed according to the method described by Xia et al. (2018). The quantitative real-time PCR(RT-qPCR) was checked with gene-specific primers to investigate the transcription levels of some genes for *cis*-abiol, gibberellins and abscisic acid biosynthetic enzymes. The RT-qPCR assay was performed using SYBR Green PCR Master Mix (Tiangen Biotech, China) for 20 µL of the reaction mixture on an IQ5 Light Cycler System (Bio-Rad, Hercules, CA, USA). Relative transcript levels were calculated as described by Zhang et al. (2019), and using the *L25* as the reference gene. Primers used for RT-qPCR are listed in **Table S3**.

### Statistical analysis

Microsoft Excel (Microsoft Corporation, USA) was used for data collection, and SPSS (version 20.0, SPSS Inc., Chicago, IL, USA) for statistical analysis of data, and RStudio (1.4.1106 RStudio, Boston USA) was used for mapping. Data were reported as mean values ± standard error (SE). Data were analyzed using one-way analysis ANOVA, and means were compared using Duncan’s multiple range test at a significance level of *p*< 0.05.

## Results

### Expression analyses of *NtCPS2* in response to abiotic and biotic stress

**Figure 1:**
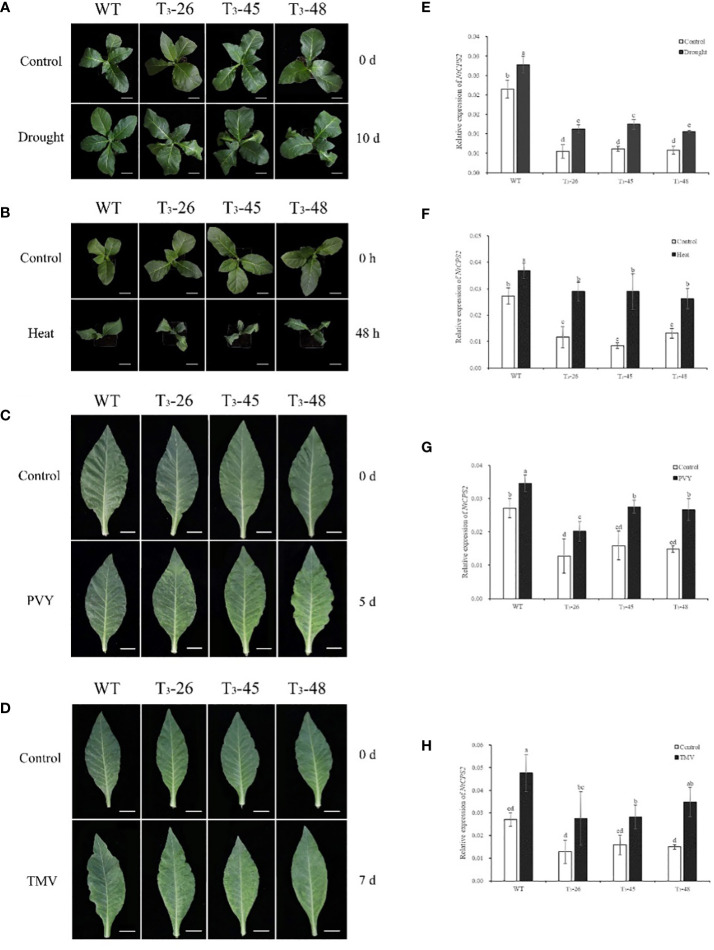
Transcript profiles of tobacco copalyl diphosphate synthase 2 (*NtCPS2*) genes in response to abiotic stress or biological stress. Ten-week-old tobacco seedlings were treated with drought, 45°C heat, PVY or TMV, the photos were taken after 6 hours of drought stress, 48 hours of heat stress, 7 days of PVY inoculation or 7 days of TMV inoculation **(A–D)**. And seedlings were sampled after photography to extract RNA, and then transcript levels of *NtCPS2* were checked by quantitative PCR (RT-qPCR) under drought stress **(E)**, heat stress **(F)**, PVY **(G)** or TMV **(H)**. In each quantitative reverse transcription quantitative PCR (RT-qPCR), the transcript levels of the tobacco reference gene *L25* in different samples were also evaluated. Three technical replicates were performed for each experiment. The data shown are the mean ± SD of three independent experiments. Statistical analysis was performed using the ANOVA test (p< 0.05) and significant differences are indicated by different letters.

To investigate the gene expression patterns of *NtCPS2* under different abiotic and biotic stresses, we examined the transcript levels of *NtCPS2* in 10-week-old tobacco seedlings exposed to drought, heat, PVY, and TMV. As shown in **Figure 1**, *NtCPS2* gene expression increased significantly within 6 hours after drought treatment, and transgenic plants wilted more than WT. After hot treatment, the expression level of the *NtCPS2* gene increased by 50% on average after 48 h, and the leaves of transgenic plants were browned earlier than WT (**Figure 1B**). After inoculation with PVY and TMV virus, the transgenic plants developed disease on days 5 and 7, respectively, but WT did not develop the disease. Meanwhile, the expression level of the *NtCPS2* gene was significantly increased. The results confirmed that the *NtCPS2* gene expression could be induced by abiotic stress and biological stress.

### Subcellular localization of *NtCPS2* protein

**Figure 2:**
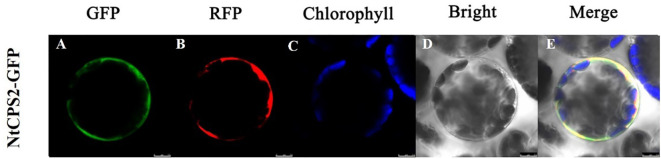
Subcellular location of *NtCPS2* in tobacco epidermal cells. **(A)** Green fluorescence of 35S::NtabDOG1L-T-GFP; **(B)** Red fluorescence of 35S::NtabDOG1L-T-GFP; **(C)** Chlorophyll fluorescence; **(D)** bright-field images; **(E)** merged images. Bars = 7.5 μm.

To monitor the subcellular location of *NtCPS2*, we fused the *NtCPS2* gene (using its CDS sequence) to the N-terminus of a GFP reporter gene, and the resulting expression cassette was transiently expressed in tobacco leaf epidermal cells. As shown in **Figure 2**, the fluorescence of the *NtCPS2*-GFP fusion protein was observed in the cell membrane.

### *NtCPS2* knock-down improves tobacco seedling growth

**Figure 3:**
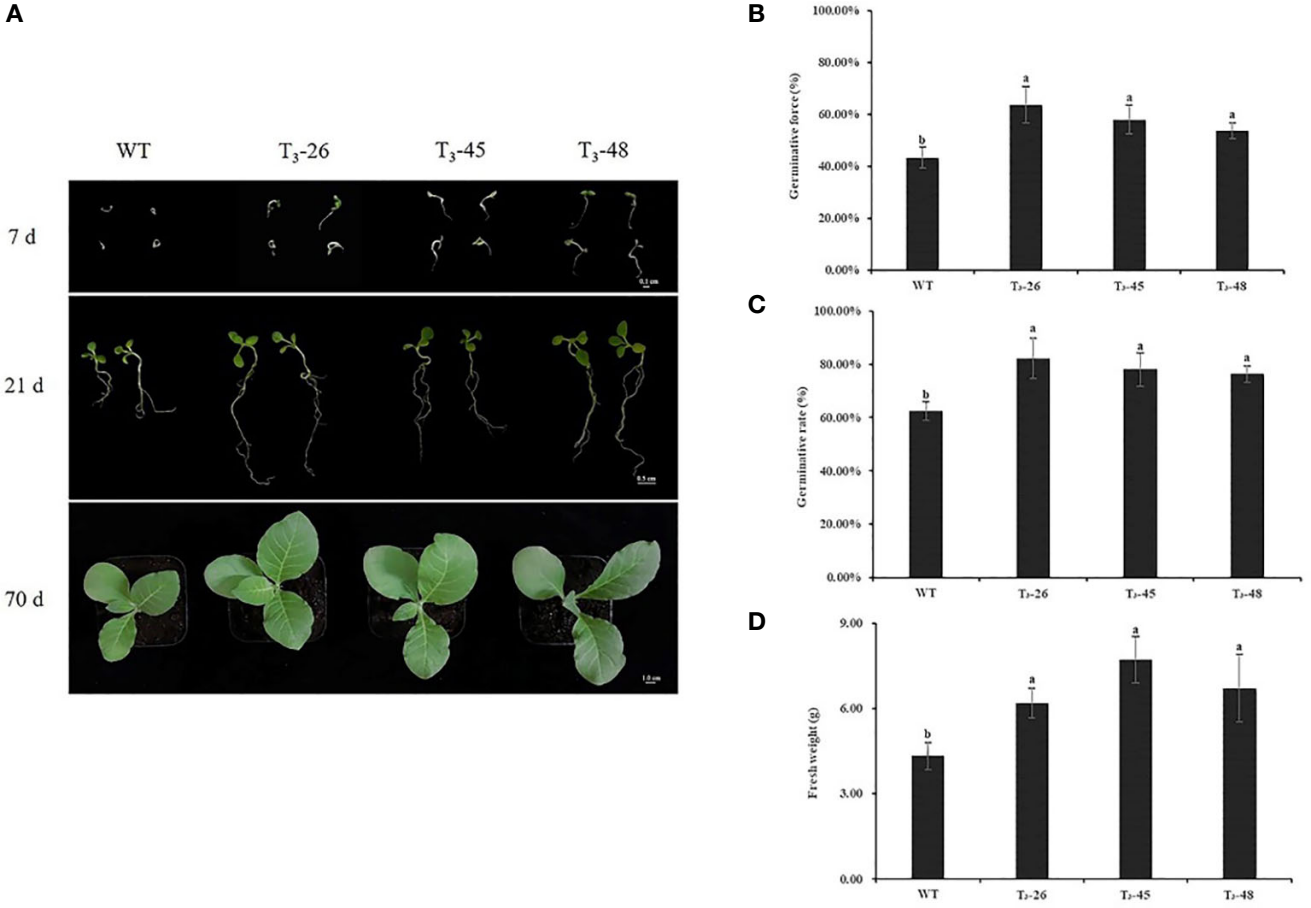
The phenotypic analyzes of *NtCPS2* transgenic plants and WT plants. **(A)** Phenotype of WT and transgenic seeds sown for 7, 21, and 70 days, respectively. **(B)** Germinative force of WT and transgenic seeds sowed for 7 days. **(C)** Germinative rate of WT and transgenic seeds sowed for 14 days. **(D)** Fresh weights of 70-day-old WT and transgenic plants. Bars = 0.1 cm, 0.5 cm or 1.0 cm. Error bars represent means ± SD. Statistical analysis was performed using the ANOVA test (p< 0.05) and significant differences are indicated by different letters. .

Under optimal growth conditions, the seed germination and seedling stage of the three transgenic lines and WT differed on the Petri dish (**Figure 3A**), the transgenic plants were larger than the WT plants (**Figure 3A**). Then, we counted the germination potential and germination rate of the tobacco seedlings on petri dish, and found that the transgenic plants had higher germination potential and germination rate than the WT plants (**Figures 3B, C**). Similarly, the transgenic lines exhibited higher seedling fresh weight than the plants from WT (**Figure 3D**).

### *NtCPS2* knock-down alters the content of GA and ABA

**Figure 4:**
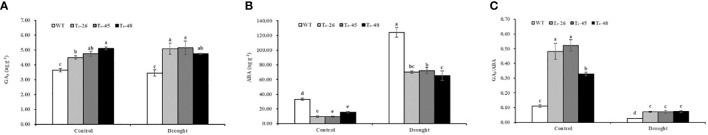
*NtCPS2* functions in endogenous hormone. **(A, B)** The content of gibberellin and abscisic acid. **(C)** Gibberellin to abscisic acid ratio. Error bars represent means ± SD. Statistical analysis was performed using the ANOVA test (p< 0.05) and significant differences are indicated by different letters.

According to the biosynthetic pathways of diterpenoids, the same substrate, geranylgeranyl pyrophosphate (GGPP), is used for the synthesis of *cis*-abienol and GA. *NtCPS2* knockdown positively affects GA synthesis. The GA3 contents in transgenic plants were 18.94%, 23.20% and 28.68% higher, respectively, under control treatment compared with that of the WT (**Figure 4A**). The GA3 content was not significantly changed under drought treatment from that of the control treatment. As shown in **Figure 4B**, the ABA contents in transgenic plants were 71.82%, 72.61% and 53.36% lower, respectively, under control treatment compared to WT. In addition, the content of ABA increased sharply under drought treatment with that of the control treatment. Meanwhile, the GA3/ABA in transgenic plants increased sharply compared with that of the WT, indicating that *NtCPS2* knockdown resulted in increased gibberellin content, and decreased abscisic acid content, and then increased the ratio of gibberellin content to abscisic acid content (**Figure 4C**). Furthermore, the changes in GA and ABA contents in transgenic plants and WT plants under drought treatment were consistent with those under control treatment.

### *NtCPS2* knock-down alters the expression of GA- and ABA- related genes

**Figure 5:**
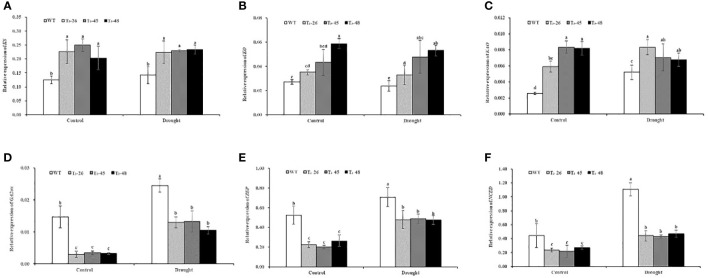
**(A)** The relative expression of ent- acid synthase gene KS. **(B)** The relative expression of ent- acid oxidase gene KO. **(C)** The relative expression of ent-acid oxidase gene KAO. **(D)** The relative expression of GA2-oxidase gene GA2ox. **(E)** The relative expression of zeaxanthin epoxidase gene ZEP. **(F)** The relative expression of 9-cis epoxycarotenoid dioxygenase gene NCED. Error bars represent means ± SD. Statistical analysis was performed using the ANOVA test (p< 0.05) and significant differences are indicated by different letters.

To further prove that *NtCPS2*-knockdown promotes GA synthesis by regulating the expression of gibberellin synthesis pathway genes, and the antagonistic effect of gibberellin and abscisic acid on transcription level, *L25* was used as a housekeeping gene to analyse the expression of 6 key genes by qRT-PCR. These genes included four gibberellin genes (ent- acid synthase gene *KS*, ent- acid oxidase gene *KO*, and ent-acid oxidase gene *KAO*, GA2-oxidase gene *GA2ox*) and two abscisic acid genes (zeaxanthin epoxidase gene *ZEP*, and 9-cis epoxycarotenoid dioxygenase gene *NCED*), which have been shown to play a key role in gibberellin and abscisic acid synthesis. As indicated in **Figures 5A-D**, the relative expression levels of *KS*, *KO* and *KAO* genes promoting gibberellic acid synthesis in these transgenic lines were higher than those in the WT under control and drought conditions. And the relative expression level of the *GA2ox* gene which inhibited gibberellic acid synthesis was lower in transgenic than in WT. Meanwhile, the relative expression levels of *ZEP* and *NCED*, which promote ABA synthesis, in transgenic lines were lower than those in the WT, especially the relative expression level of *NCED* in transgenic lines was 59.58% lower than that in the wild type under drought conditions (**Figures 5, F**). These data indicate that *NtCPS2* knockdown regulates the expression of gibberellin and abscisic acid-related genes, and significantly affects the expression of abscisic acid-related genes under drought stress.

### *NtCPS2* knock-down increased the stomatal aperture and water loss of the transgenic plants

**Figure 6:**
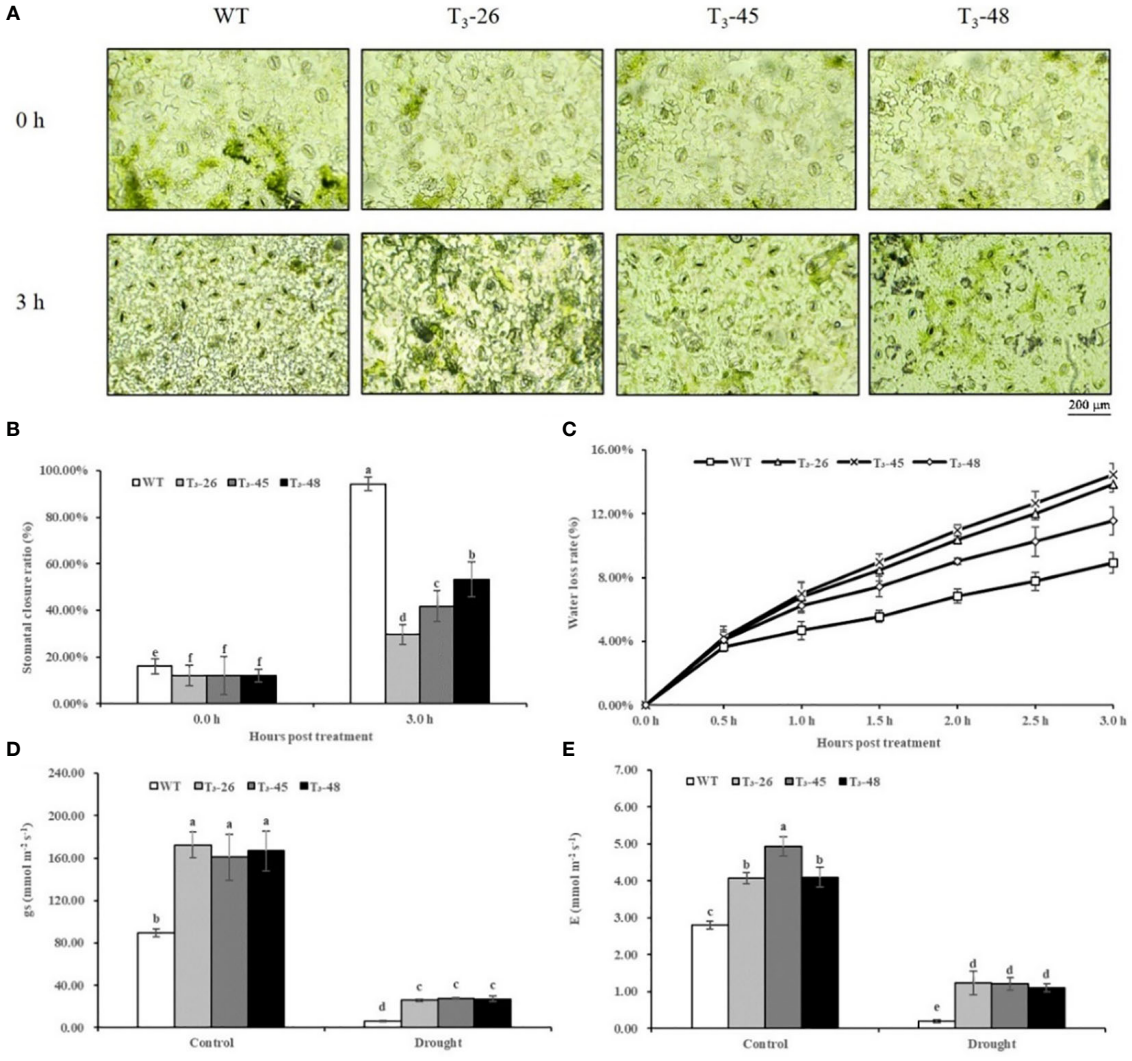
*NtCPS2* functions in drought tolerance. **(A)** Stomata of WT and transgenic plants under desiccation treatment. Scale bar =200 μm. **(B)** Ratio of stomata of WT and transgenic lines after dehydration. **(C)** Water loss rates of leaves detached from WT and transgenic lines. **(D)** Stomatal conductance (gs) of WT and transgenic lines after 6 h of drought treatment. **(E)** Transpiration rate **(E)** of WT and transgenic plants after 6 h of drought treatment. Error bars represent means ± SD. Statistical analysis was performed using the ANOVA test (p< 0.05) and significant differences are indicated by different letters.

Next, we investigated the functions of *NtCPS2* in drought stress tolerance using transgenic tobacco. Water loss rate and stomatal aperture are often used as indicators of drought tolerance in plants. The stomatal openings of the *NtCPS2* transgenic *NtCPS2* plants were significantly larger under drought stress at room temperature than those of the WT (**Figure 6A**). And after 3 hours of dehydration stress, the stomatal closure rate of *NtCPS2* transgenic plants was significantly smaller than that of WT (**Figure 6B**). Similarly, water loss from the detached leaves was recorded every half hour in both *NtCPS2* transgenic lines and the plants from WT. As shown in **Figure 6C**, the three transgenic plants had higher water loss rates than the plants from WT.

To further detect stomatal closure and water loss of leaves, we tested stomatal conductance and transpiration rate of leaves, and found that after drought treatment for 6 hours, stomatal conductance and transpiration rate of the *NtCPS2* transgenic plants were significantly higher than those of WT (**Figures 6D, E**). These tests showed that the decreased expression of *NtCPS2* gene was not good for plant leaf water retention, resulting in the lower drought resistance of *NtCPS2* transgenic plants than WT.

### *NtCPS2* knock-down reduces the antioxidant capacity of transgenic tobacco plants

To uncover the possible physiological mechanisms underlying the enhanced drought tolerance of *NtCPS2*-knockdown plants, we measured the levels of hydrogen peroxide (H_2_O_2_) and the activities of several antioxidant enzymes in *NtCPS2*-knockdown plants and WT plants grown under optimal conditions and drought.

**Figure 7:**
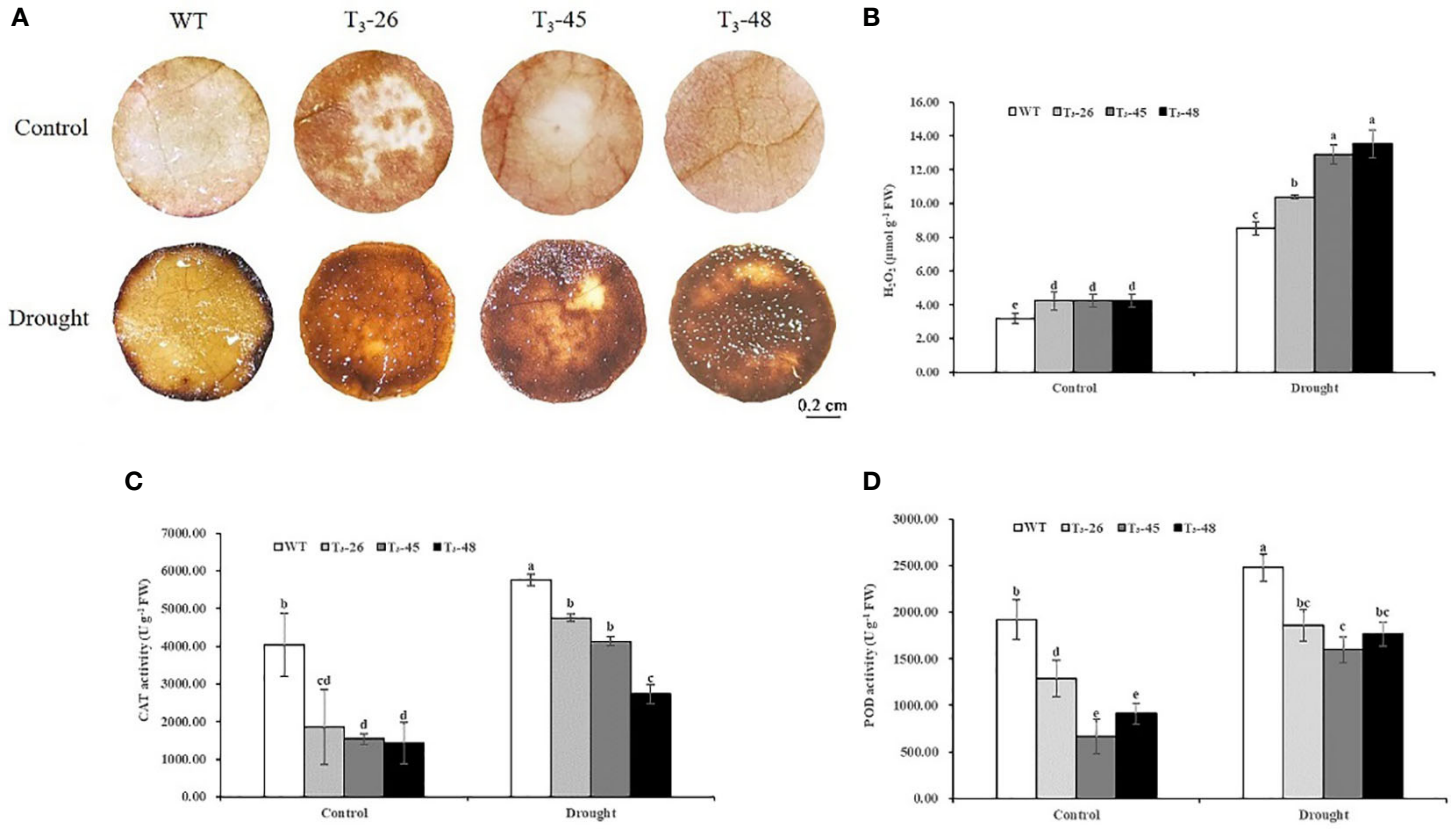
*NtCPS2* functions in antioxidant capacity. After 6 h of drought treatment, WT and transgenic plants were stained with DAB **(A)** and subjected for H_2_O_2_ content determination **(B)**. The activity levels of antioxidant enzymes CAT **(C)**, POD **(D)** in three transgenic plants and WT plants were treated with drought for 6 h. Scale bar =0.2 cm. Error bars represent means ± SD. Statistical analysis was performed using the ANOVA test (p< 0.05) and significant differences are indicated by different letters.

To confirm the ability of the transgenic tobacco plants to scavenge ROS, the accumulation of ROS was determined under optimal growth conditions and under drought stress. There was a difference in H_2_O_2_ accumulation between WT and transgenic seedlings (**Figure 7A**). After drought treatment for 6 hours, H_2_O_2_ accumulation in transgenic lines was higher than WT, observed as brown (DAB staining) pigments (**Figure 7A**). To quantify this difference, H_2_O_2_ content was measured in whole tobacco seedlings with or without drought stress. Under optimal growth conditions, H_2_O_2_ content was slightly higher in the transgenic line than in WT (**Figure 7B**). After 6 hours of drought, three transgenic lines contained significantly more H_2_O_2_ than WT, especially T_3_-45 and T_3_-48 (**Figure 7B**).

Next, we evaluated the activities of two antioxidant enzymes, guaiacol peroxidase (POD) and catalase (CAT) in the transgenic and WT plants (**Figures 7C, D**). The results showed that POD and CAT activities significantly increased after drought stress, however, the increase in transgenic plants was less than that in WT plants (**Figures 7C, D**).

These results suggest that *NtCPS2*-knockdown increases the accumulation of ROS by reducing the activities of several antioxidant enzymes.

### *NtCPS2* knock-down increases the membrane permeability of transgenic tobacco plants

**Figure 8:**
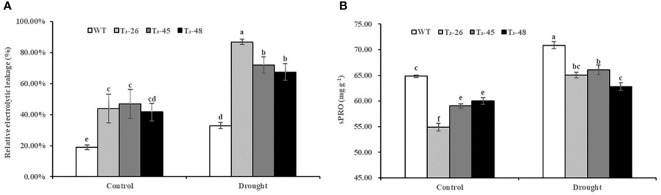
*NtCPS2* functions in membrane permeability. **(A)** Comparisons of relative electrolyte leakage rates in the transgenic lines and WT. **(B)** The content of soluble protein (sPRO) in the transgenic lines and WT. Error bars represent means ± SD. Statistical analysis was performed using the ANOVA test (p< 0.05) and significant differences are indicated by different letters.

Furthermore, to further confirm the status of the membrane system in transgenic plants, we examined the relative electrolytic leakage and soluble protein content (**Figures 8A, B**). The relative electrolytic leakage in these transgenic lines was about 25% and 42% higher than those in WT plants under suitable and drought conditions, respectively. (**Figure 8A**). Furthermore, the soluble protein content was significantly lower in the transgenic lines compared to WT, especially after treatment with drought stress (**Figure 8B**). These results evidenced that the increased membrane permeability caused by *NtCPS2* knockdown is due to the decreased sPRO content, which reduces the tolerance to drought stress.

## Discussion

To date, most of the studies have been focused on the roles of *CPS2* genes in the important synthesis of diterpene-diol *cis*-abienol processes including in balsam fir (*Abies balsamea*), tobacco (*Nicotiana tabacum; family Solanaceae*) and Bolivian sunroot (*Polymnia sonchifolia; family Asteraceae*) (Cheng et al., 2002). Recently, through transcriptome analyses, we have discovered that *NtCPS2* genes is involved in the synthesis of GAs (He et al., 2021), reducing GAs levels or signaling promotes plant tolerance to environmental stresses, including drought (Nir et al., 2014). In this study, *NtCPS2* had strong responses to drought, heat, PVY, and TMV treatments. Meanwhile, phenotypes of *NtCPS2* knockdown lines and WT plants were significantly different under abiotic and biological stresses. In addition, we report phenotypic analyses of molecular and physiological responses of the *NtCPS2* knockdown plants under drought stress. Our results have demonstrated that *NtCPS2* knockdown reduces drought stress tolerance by changing plant hormone levels, breaking antioxidant metabolism and membrane permeability.

Both *cis*-abienol and GAs are diterpene compounds. GGPP is used as a precursor to generating *cis*-abienol and *ent*-kaurene through terpene synthases (TPSs) catalysis, and then *ent*-kaurene is treated by cytochrome P450 monooxygenases (P450s) and 2-oxoglutarate-dependent dioxygenases (2ODDs) generates GAs (Smith et al., 1998; Okada et al., 2000; Achard et al., 2006; Shi et al., 2006). After the gene *NtCPS2* encoding terpene synthase was edited, the content of *cis*- abienol decreased while GAs content increased significantly, so it was speculated that the decrease of *cis*-abienol content may reduce the competition for common precursor GGPP (Zhang et al., 2020) and lead to the increase of GAs content (He et al., 2021a). In addition, this study found that editing the *NtCPS2* gene affected related genes in the GAs synthesis pathway, thus increasing GAs content, but at the same time, after editing the *NtCPS2* gene, the ABA content and related gene expression levels of common precursors with *cis*-abienol alcohol and GAs were significantly reduced. Therefore, the effect of editing the *NtCPS2* gene on ABA content is different from that of GAs, and the decrease in ABA content may be caused by its antagonism with GAs (Lin et al., 2020). Zhou et al. (2010) found that exogenous application of different concentrations of GA3 reduced the ABA content of Guar beans, and the results of this study were similar. Studies have also shown that gas-induced Ca^2+^/calmodulin signaling regulates hydrolase synthesis and secretion, while ABA blocks the expression of hydrolase, which may explain the antagonism between GA and ABA (Sun and Guble, 2004; Yu and Helen, 2011).

**Figure 9:**
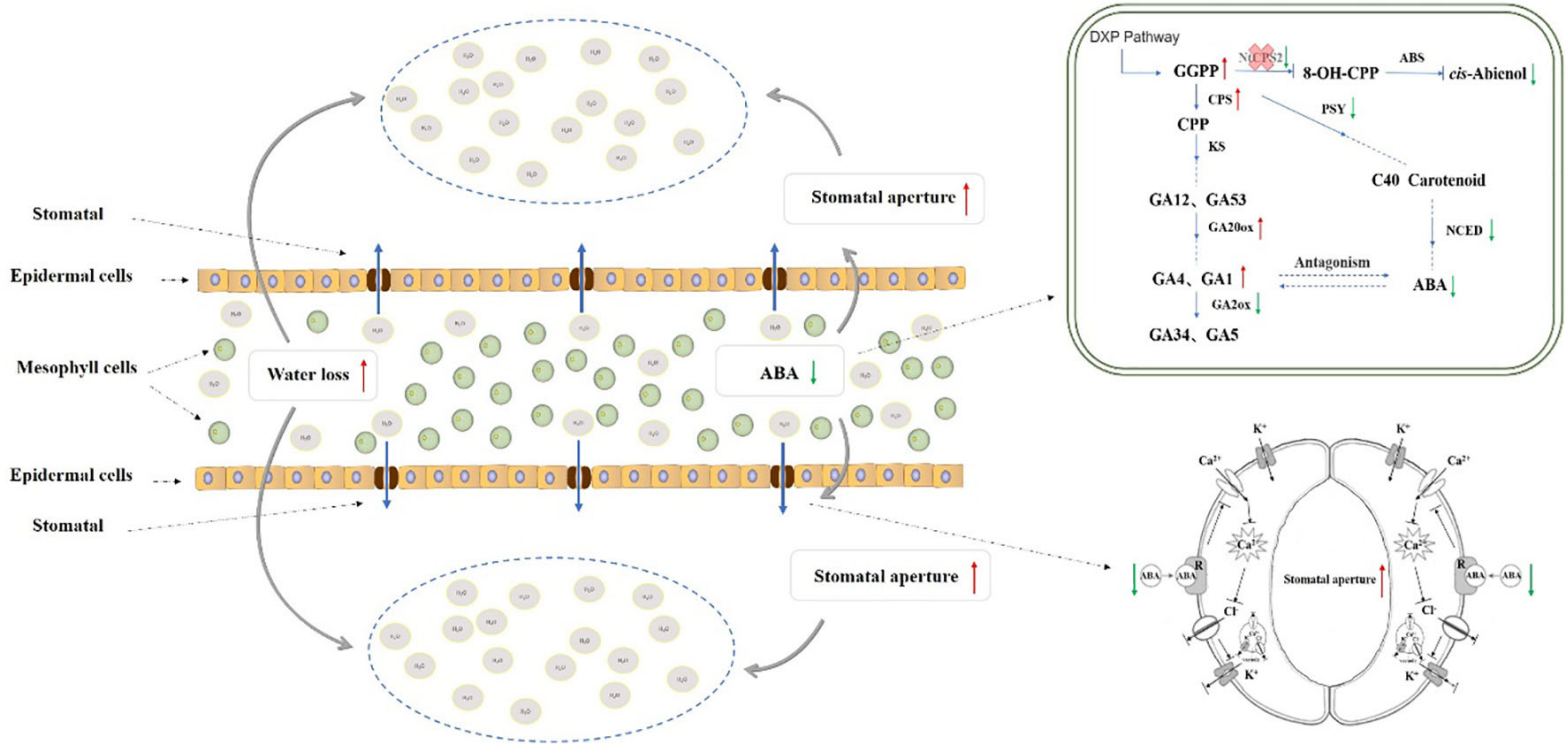
Metabolic network of the effect of *NtCPS2* gene on drought resistance in tobacco.

It is well documented that the plant hormone ABA plays an important role in the reaction of plants to drought (Vishwakarma et al., 2017). The decrease of ABA content in *NtCPS2*-knockdown tobacco resulted in the decline of stomatal closure, an increase of stomatal conductance and reduction of drought resistance. Wang et al. (2004) showed a significant negative correlation between ABA content and stomatal opening, and the results of this study were similar. Meanwhile, Tal et al. (1972) also showed that the stomatal aperture of plant mutants with too low endogenous ABA level significantly increased. Živanović et al. (2021) found that the two ABA levels formed different tomato genotypes WT and *flacca* mutant tomato had different responses to drought. Compared with WT, ABA content in leaves of the mutant tomato decreased by about 25%, resulting in more obvious stomatal opening and reduced drought resistance. The results of our study were similar to Živanović et al. (2021), suggesting that the decrease of ABA content indirectly led to the sensitivity of plants to drought stress, and it was speculated that the increase of stomatal conductance caused by the decrease of ABA content could lead to the increase of water loss and the decrease of drought resistance of leaves (Virlouvet and Fromm, 2014; Fleta-Soriano et al., 2015) In conclusion, after editing the *NtCPS2* gene, the relative expression level of the *NtCPS2* gene in *NtCPS2*-knockdown tobacco decreased, and the content of GGPP increased. Meanwhile, the relative expression level of the positive regulation gene of GAs synthesis increased, and the relative expression level of negative regulation gene decreased. These physiological molecular changes lead to an increase in GAs content and a decrease in ABA content of *NtCPS2*-knockdown tobacco, thus reducing the stomatal closure rate. As a result, the water diffused from mesophyll tissue to the outside environment through stomata increases, thus increasing the sensitivity of plants to drought stress. The metabolic synthesis network is shown in **Figure 9**.

Plants usually cope with drought stress in three ways: shortening the life cycle or improving developmental adaptability, increasing water absorption and reducing water loss, regulating osmosis, antioxidant capacity and drought tolerance (Yue et al., 2006). Our results suggest that changes in several mechanisms in *NtCPS2*-knockdown plants jointly show increased sensitivity to drought stress. In this study, we first found that tobacco with *NtCPS2*-knockdown significantly improved seedling growth, seed germination and seedling fresh weight (**Figure 3**). The higher seed germination and fresh weight of the transgenic plants (**Figure 3**), could absorb more water under water deficit conditions thus increasing drought stress. Secondly, high stomatal opening leads to continuous wilting of the leaves (Tal and Imber, 1972). In the process of dehydration, the stomatal closure rate of *NtCPS2*-knockdown plants was significantly lower than that of WT plants, while the stomatal conductance and transpiration rate were increased (**Figure 6**), suggesting that the transgenic plants lose more water under drought conditions. Third, the relative electrolyte loss under drought stress was higher in transgenic plants than in WT, and sPRO content was lower than in WT (**Figure 8**). In addition, the activities of POD, and CAT were decreased in the transgenic plants under drought conditions (**Figures 7C, D**) and H_2_O_2_ accumulation was enhanced (**Figure 7B**). Electrolyte losses are commonly used as indicators of membrane damage and plant resistance (Tsikas, 2017; Demidchik et al., 2014). Antioxidant enzymes can remove harmful substances and reduce membrane damage in an unfavourable environment (Chen et al., 2017; Khan et al., 2019). The results showed that *NtCPS2*-knockdown tobacco exhibited lower drought resistance.

The advantages and limitations of the present study. In previous studies we used CRISPR/Cas9 gene editing technology to knock down *NtCPS2*. A CRISPR/Cas9 *NtCPS2* expression vector was constructed with the high aroma plant 8306 and *NtCPS2* knockout transformed plants were obtained. High-throughput RNA sequencing (RNA-seq) technology was used to compare the expression profiles of mutant and 8306 plants. The sequencing results were validated by fluorescence quantitative polymerase chain reaction (PCR) and relevant physiological and biochemical assays were performed, which revealed that the *NtCPS2* gene not only affected the lysobarbital-like diterpene metabolic pathway, effectively reducing the content of cis-cryptoxanthin, but also affected the gibberellin terpene metabolism, increased the content of gibberellin and thus increased the height of the plants.

Based on the results of previous studies, the pure editorial line 8306 was subjected to biotic and abiotic stresses and the function of the *NtCPS2* gene was investigated. In this study, although biotic (drought, high temperature) and abiotic stresses (PVY, TMV) were applied to 8306 wild-type and pure editorial lines, the focus of the experiment was on drought stress, and several other stresses were used only as an auxiliary proof of the results.

Taken together, these data indicate that *NtCPS2* negatively affects tobacco drought tolerance at least in part, through the decrease in ABA content and antioxidant capacity induced by the up-regulation of GAs gene expression, as well as the down-regulation of ABA gene expression following the decrease in relative *NtCPS2* gene expression. Our results provide valuable information for the potential application of *NtCPS2* in genetically improving drought of crops and gene pleiotropy of *NtCPS2*.

## Data availability statement

The raw data supporting the conclusions of this article will be made available by the authors, without undue reservation.

## Author contributions

SX and LH designed the study, WH, KC, BL, CZ, KX and WL performed the experiment. SX analyzed the data and drafted the manuscript. All authors contributed to the article and approved the submitted version.

## Funding

The funder China National Tobacco Corporation Henan company, Guizhou Tobacco Corporation Guiyang company, Hunan Tobacco Corporation Changsha Company, Guizhou Tobacco Corporation Tongren company was not involved in the study design, collection, analysis, interpretation of data, the writing of this article or the decision to submit it for publication. All authors declare no other competing interests. This research was funded by China National Tobacco Corporation Henan company (2021410000240021); Fujian Tobacco Corporation Nanping Company (2021350700240072); Guizhou Tobacco Corporation Guiyang company (2020-07); Hunan Tobacco Corporation Changsha Company (21-23A04); Guizhou Tobacco Corporation Tongren company (202101).

## Conflict of interest

Author SX, WH, and KC are employed by Henan Agr Univ, Coll Tobacco Sci, Natl Tobacco Cultivat & Physiol & Biochem Res Ctr, Scientific Observation and Experiment Station of Henan, Ministry of Agriculture. Author BL is employed by China Tobacco Zhejiang Industry Co, Ltd. Author CZ, KX, and WL are employed by Fujian Tobacco Corporation Nanping Company. Author LH is employed by College of Agronomy, Sichuan Agricultural University & Sichuan Engineering Research Center for Crop Strip Intercropping System & Key Laboratory of Crop Ecophysiology and Farming System in Southwest.

## Publisher’s note

All claims expressed in this article are solely those of the authors and do not necessarily represent those of their affiliated organizations, or those of the publisher, the editors and the reviewers. Any product that may be evaluated in this article, or claim that may be made by its manufacturer, is not guaranteed or endorsed by the publisher.
