# Supplementary figures and images for: Knockdown of NtCPS2 promotes plant growth and reduces drought tolerance in Nicotiana tabacum
Source: Front Plant Sci. 2022 Nov 8;13:968738. doi: 10.3389/fpls.2022.968738 (PMC9679219; doi:10.3389/fpls.2022.968738)

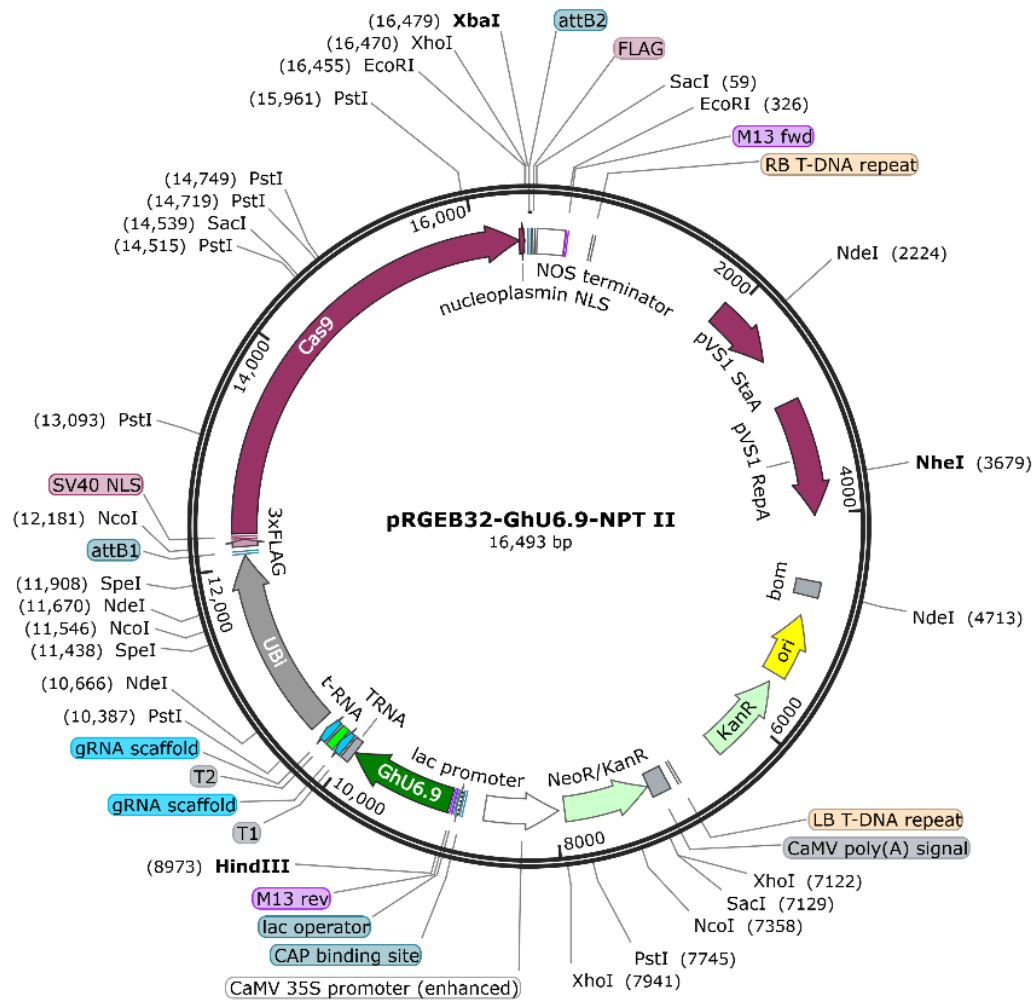

**Figure S1. CRISPR/Cas9 vector composition (promoter used).**

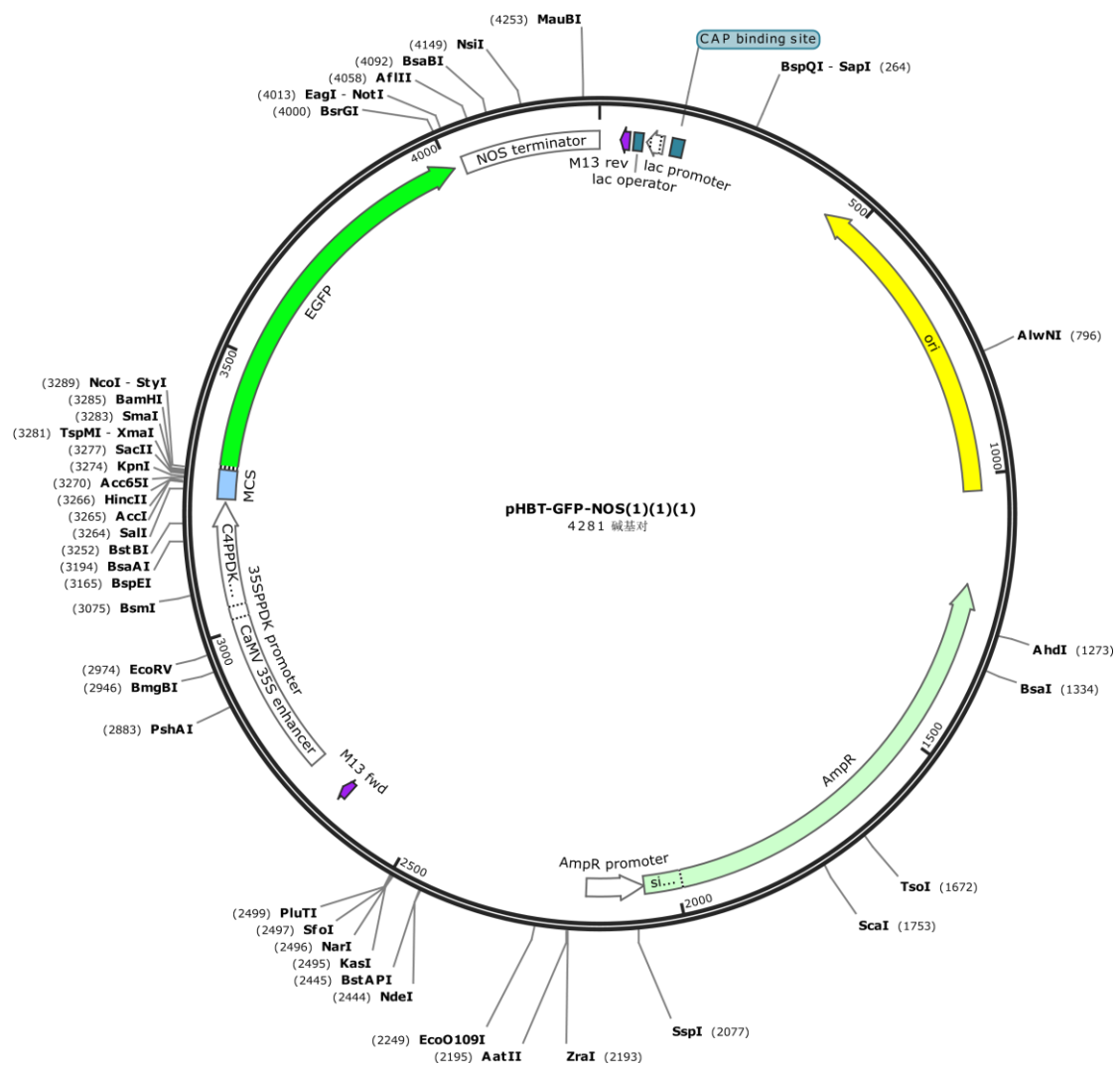

**Figure S2. GFP proteins vector map.**

Supplement: Supplementary file 1 [file DataSheet_1.pdf]
