# Supplementary material for: Knockdown of NtCPS2 promotes plant growth and reduces drought tolerance in Nicotiana tabacum
Source: Front Plant Sci. 2022 Nov 8;13:968738. doi: 10.3389/fpls.2022.968738 (PMC9679219; doi:10.3389/fpls.2022.968738)
Supplement: Supplementary file 2 [file DataSheet_2.pdf]

Supplementary Table 1. The sequences of target sites

Target site	Sequence (5'—3')
KN48-T1	GGAAACCCAAGCTGTGTCATAGG
KN48-T2	CCCCATGAACCATCAGAAAGTTG

Supplementary Table 2. The primers for PCR amplification

Primers	Sequence
pRGEB32-7s（P1）	AAGCATCAGATGGGCAAACAAAGCACCAGTGGTCTA
inf pRGEB32-7s	AAGCATCAGATGGGCAAACAAA
KN48-T1 as（P2）	ATGACACAGCTTGGGTTTCCTGCACCAGCCGGGAAT
KN48-T1 s（P3）	GGAAACCCAAGCTGTGTCATGTTTTAGAGCTAGAAAT
KN48-T2 as（P4）	CATGAACCATCAGAAAGTTGTGCACCAGCCGGGAAT
KN48-inf T2 as	TTCTAGCTCTAAAACCATGAACCATCAGAAAGTTG

Supplementary Table S3 Sequences of fluorescence quantitative primers(5′→3′)

Gene	Forward primer	Reverse primer
L25	CCCCTCACCACAGAGTCTGC	AAGGGTGTTGTTGTCCTCAATCTT
CPS2	ACTCGTGTCTCTTGGTACT	CACACTCCAATTTAAGCCG
KS	AAGTGTGAATTAGGAATGTCGT	ATGCCACTTCAAATCCGATAG
KO	TATCCCGGTAACTATCGTCTTG	ACATACCTCGCTCTGCTAA
KAO	AATTGGATTGATGGTGAGCA	CACCTGGTGGCAATGATAA
GA2ox	AGCCATTAAATTCTTCTCCTCT	TACGACCAATCTGTTTATTGCC
ZEP	TCGTGACATCTATGATAGACCG	CCTTGACCCAAATTAGGCTG
NCED	ACTCTTCTTCTACAACACATCC	TTGTTAAGGCACTTTCCACG
